# Monorail technique for retrieval of a proximally migrated pancreatic stent

**DOI:** 10.1016/j.igie.2026.01.020

**Published:** 2026-02-06

**Authors:** Karthik Chandrasekaran, Hiroyuki Isayama, Bashar Qumseya, Peter V. Draganov

**Affiliations:** 1 Department of Gastroenterology, Hepatology and Nutrition, University of Florida, Gainesville, Florida, USA; 2 Department of Gastroenterology, Graduate School of Medicine, Juntendo University, Tokyo, Japan

Proximal migration of pancreatic stents (PSs) is a rare but challenging adverse event of endoscopic retrograde cholangiopancreatography (ERCP). Various extraction methods, such as the gooseneck snare device, the zipline technique, and the turned guidewire looping technique, have been described for the removal of migrated PSs. However, they are not applicable in all clinical situations, and some require the use of specialized devices (ie, Soehendra stent retriever [Cook Endoscopy, Winston-Salem, NC, USA], pancreatoscopy). We present a monorail technique for the retrieval of a migrated PS using standard devices.

A 34-year-old woman underwent endoscopic ampullectomy with placement of a 5 F/9-cm single internal flange/distal pigtail Zimmon PS (Cook Endoscopy). Upon her return for stent removal, the stent was grasped with a snare; however, it inadvertently fractured into 2 pieces with upstream migration of the inner fragment into the pancreatic duct (PD) (Fig. 1). Retrieval attempts with the extraction balloon were unsuccessful and resulted in further upstream migration.

**Figure 1:**
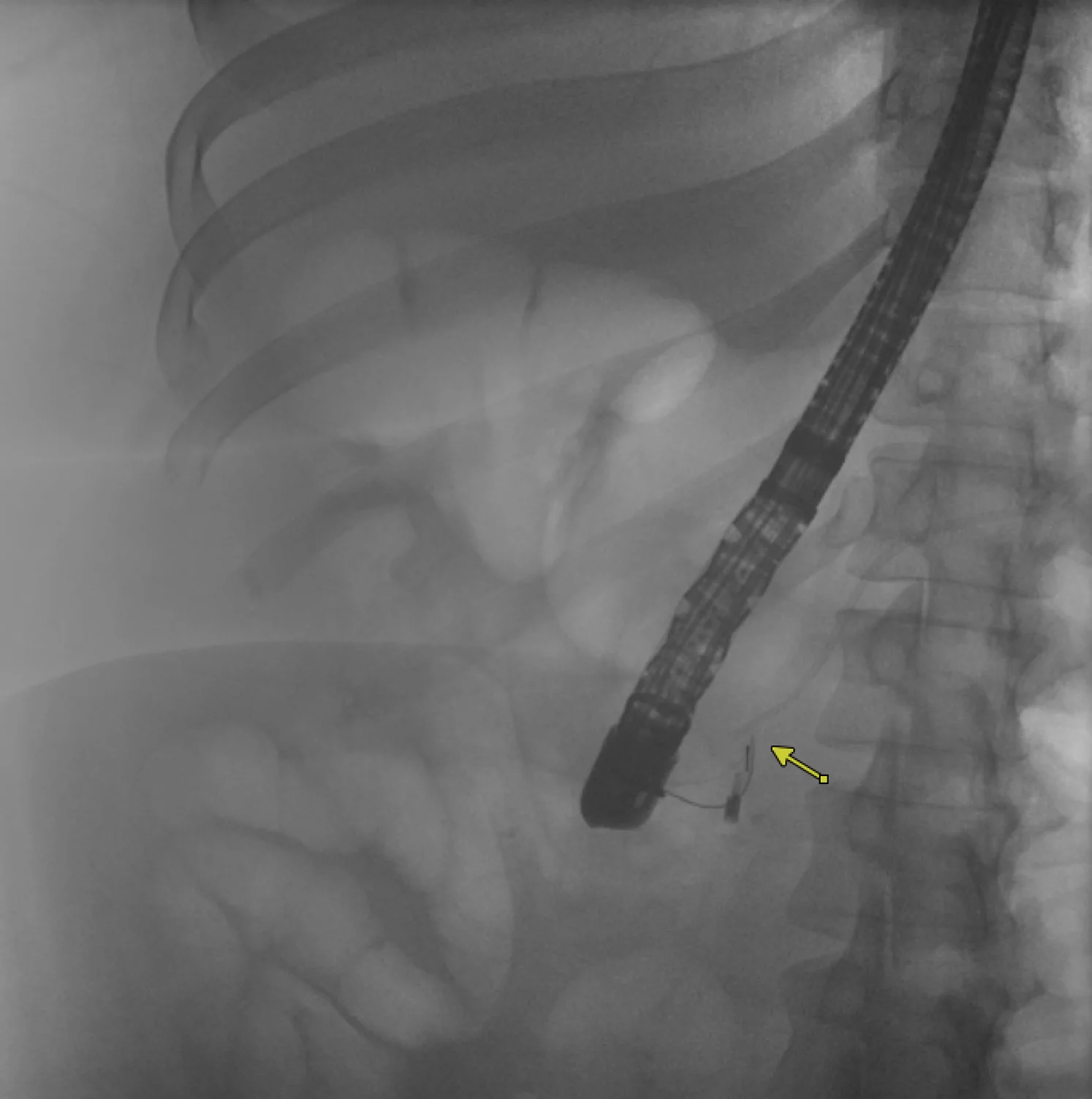
Fluoroscopic image depicting cannulation of the pancreatic duct with an *arrow* depicting a fractured plastic stent.

A 450-cm, 0.025-inch VisiGlide straight-tip guidewire (Olympus America, Center Valley, Pa, USA) was advanced via an ERCP cannula through the stent lumen (Fig. 1). A 10-mm Exacto cold snare (STERIS Corporation, Mentor, Ohio, USA) was first fully opened and fed over the back end of the guidewire (Fig. 2). The snare was then almost fully closed, leaving a small wire loop of the snare outside the plastic snare sheath in order to use the ERCP wire as a monorail guide (Fig. 3). It is important to gently close the snare snugly over the wire, but not completely over the wire, because the wire coating could be damaged or cut by the snare, leading to further issues. The snare was advanced into the PD over the guidewire until the plastic sheath of the snare abutted the distal (downstream) end of the stent. The snare was opened and advanced over the stent. The snare was then closed, capturing the migrated stent. The snare, stent, and wire were then simultaneously extracted from the PD initially under fluoroscopic guidance until the stent was removed intact from the patient (Fig. 4). The patient received 100 mg of rectal indomethacin and 2 L of intravenous lactated ringer and did not experience post-ERCP pancreatitis.

**Figure 2:**
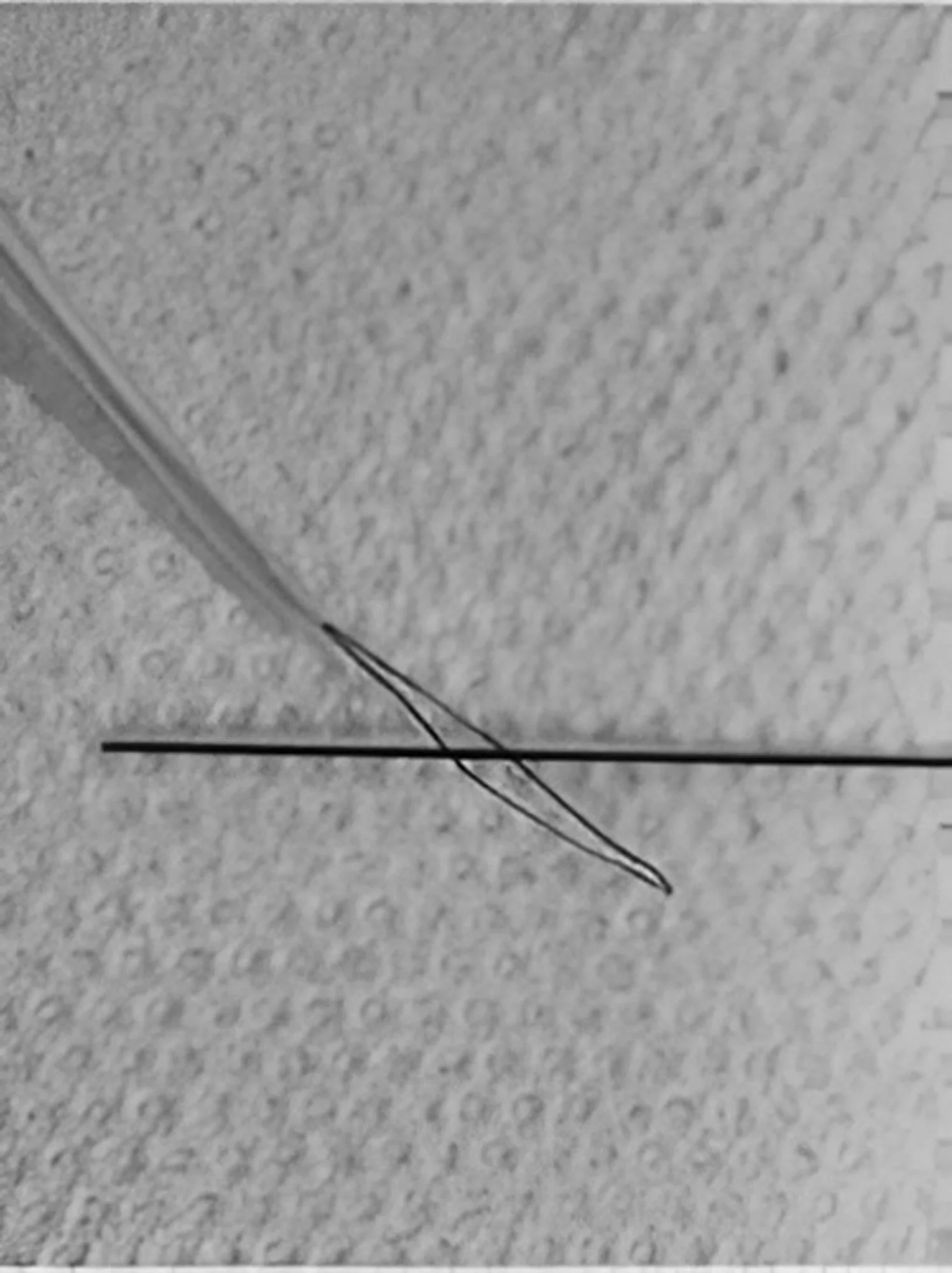
Feeding of the guidewire through a 10-mm Exacto cold snare (STERIS Corporation, Mentor, Ohio, USA).

**Figure 3:**
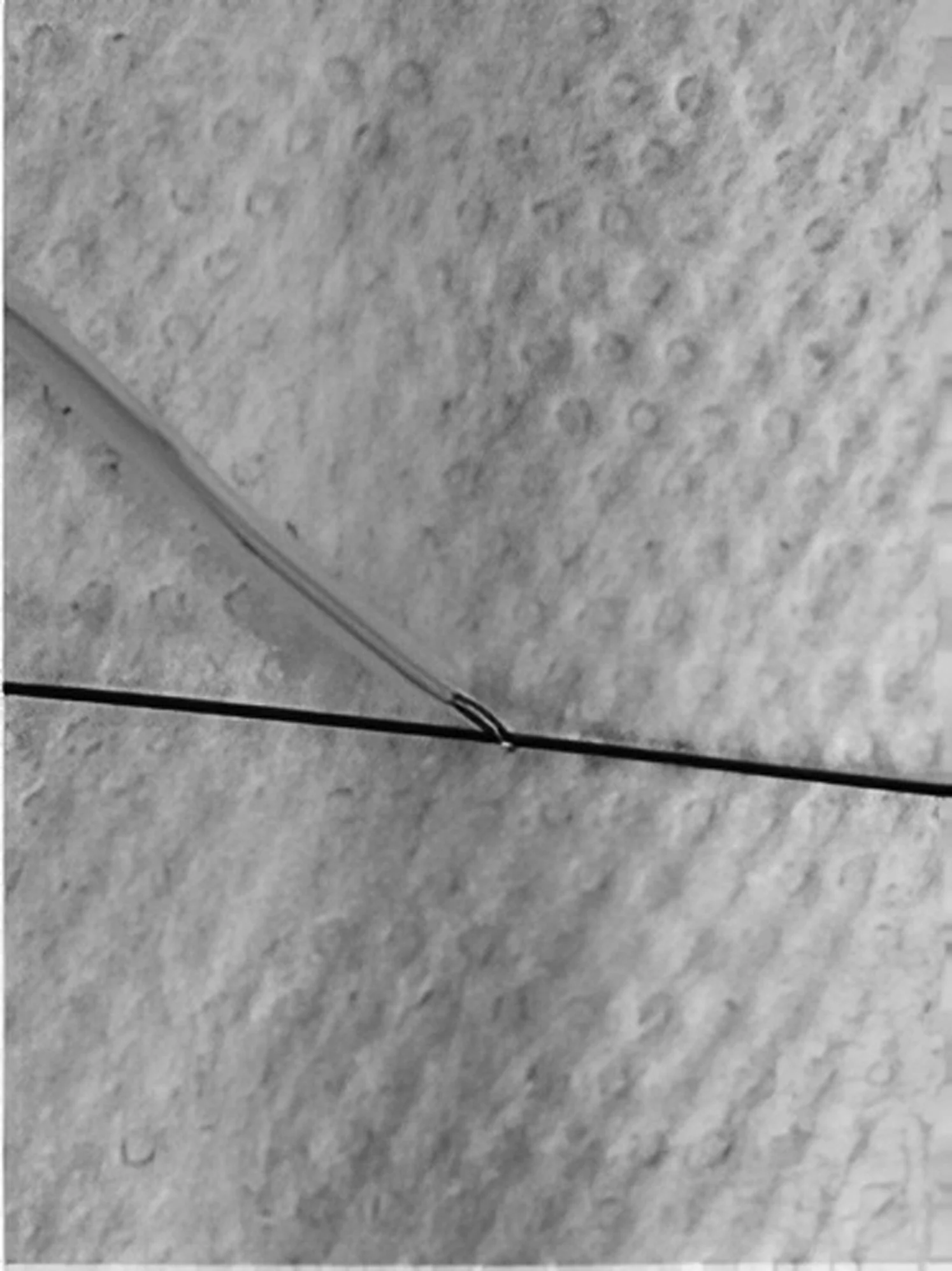
Creation of monorail guide with snare loop over guidewire.

**Figure 4:**
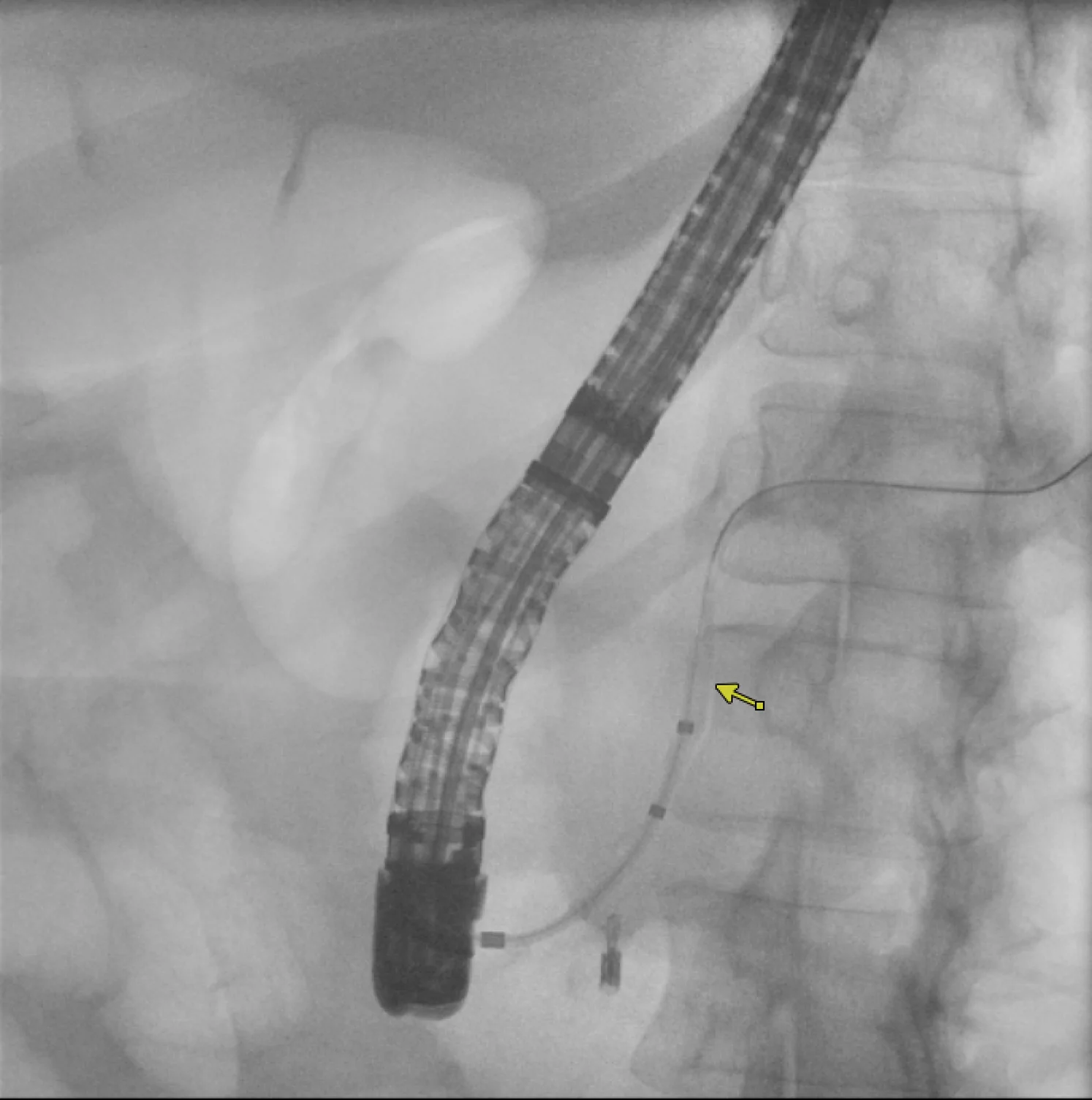
Fluoroscopic imaging depicting retrieval of a migrated stent with a snare loop (*arrow*) closed over the downstream end of the stent.

The described technique uses routinely available devices and is feasible in patients with normal-sized PD (where typically the use of stone extraction baskets or pancreatoscopy is not feasible). The 10-mm cold snare contains a small-diameter outer sheath that can be preferentially maneuvered into the PD over the ERCP guidewire in a monorail fashion. The limitation of our technique is that the guidewire must be advanced through the lumen of the migrated stent. Although this can be challenging, it is possible in patients with normal-sized PD, as the guidewire preferentially tracks into the stent lumen. The monorail technique expands our armamentarium for treating this challenging ERCP adverse event.

## PATIENT CONSENT

Written informed consent was obtained from the patient for the publication of this case description and any accompanying images. All patient-identifying information has been removed, and the material has been anonymized to protect patient privacy and confidentiality. This article describes a single patient and did not constitute research requiring Institutional Review Board approval.

## Disclosure

The following authors disclosed financial relationships: H. Isayama Consultant for SB-Kawasumi and Olympus; research support from Fujifilm, Boston Scientific, Century Medical, and Japan Lifeline; speaker for SB-Kawasumi, Fujifilm, Boston Scientific, Century Medical, Piloax Medical Devices, Kaneka Medix, and Japan Lifeline. P. V. Draganov: Consultant for Olympus, Fujifilm, Boston Scientific, Cook Medical, Medtronic, Merit, and Microtech. All other authors disclosed no financial relationships.

